# Predictors of Parental Accommodation and Response Treatment in Young Children With Obsessive-Compulsive Disorder

**DOI:** 10.3389/fpsyt.2021.737062

**Published:** 2021-11-16

**Authors:** Ángel Rosa-Alcázar, Ana I. Rosa-Alcázar, José Luis Parada-Navas, Pablo J. Olivares-Olivares, Encarnación Rosa-Alcázar

**Affiliations:** ^1^Department of Psychology, Catholic University of Murcia, Murcia, Spain; ^2^Department of Personality, Assessment and Psychological Treatment, University of Murcia, Murcia, Spain; ^3^Department of Education, University of Murcia, Murcia, Spain

**Keywords:** obsessive-compulsive disorder, young children, parental accommodation, externalizing symptoms, predictors

## Abstract

**Background:** Cognitive–behavioral family-based treatment (CBFT) is the treatment standard in very young children with obsessive–compulsive disorder (OCD), which includes the same core components of cognitive–behavioral therapy (CBT) with significant family involvement. Although the latter reports high rates of remission, some children do not improve with treatments. Therefore, it is necessary to identify possible moderating variables such as comorbidity, severity of disorder, years of onset, parental anxiety, and parental accommodation. This study has two main aims: (1) to propose a predictive model on family accommodation (father and mother), taking into account variables related to the children (severity of obsessive–compulsive responses, internalizing and externalizing symptoms, and comorbidity) and with the parents before intervention (worry, accommodation of one parental member over the other) and (2) to examine the mediating role of externalizing symptoms and mother's accommodation in the relation between initial severity and improvement of severity of obsessive–compulsive responses in children aged 5–8 years.

**Methods:** Participants comprised 56 children with OCD [mean = 6.61 (SD = 0.76)] and their parents; 79% of the sample was men. Treatment was implemented by two clinicians specialized in OCD (>15 years of experience). Clinicians were trained to administer CBT protocol in the same way. They were doctors of clinical psychology and researchers at the OCD.

**Results:** Mother's accommodation was associated with child variables (Child Behavior Checklist–Externalizing and Initial Severity, Children's Yale–Brown Obsessive–Compulsive Scale). Father's accommodation could be explained by parent variables (mother's accommodation and worry). Simple mediation model tested using the SPSS macro PROCESS supported the relation of the initial severity of symptoms with that following intervention, through the simple indirect effect of externalizing symptoms of the child.

**Conclusions:** Comorbidities with externalizing symptoms, father's worry, and mother's accommodation were variables that should be controlled in treatment of pediatric OCD.

## Introduction

Pediatric obsessive–compulsive disorder (OCD) is a debilitating psychological condition associated with deterioration of functioning in social, scholastic, and family activities (1). Several studies report early-onset OCD, indicating that for various reasons children are often underdiagnosed. Some reasons are that the child/adolescent hides the worries and rituals due to the low intensity of the discomfort. In addition, parents often believe that worries and rituals are fleeting (2, 3). On the other hand, a high percentage of adults with OCD admit to having begun suffering alterations in childhood or adolescence (2).

Recent studies have estimated the prevalence of OCD among children to be close to 3% (4). OCD in childhood/adolescence usually occurs together with other comorbidities, increasing the degree of discomfort and complicating prognosis and response to treatment (5). Several studies have found high rates of comorbidity, with percentages ranging from 50 to 80% (6). The most common associated disorders are anxiety disorders (26–75%), mood disorders (25–62%), tics (20–30%), attention-deficit/hyperactivity disorder (10–50%), and disruptive behavior (18–33%) (2, 7–9).

Treatment options for children with OCD include cognitive–behavioral therapy (CBT), pharmacotherapy, or both (2, 10). The American Academy of Child and Adolescent Psychiatry Committee on Quality Issues (2) considers that the standard of care in very young children with OCD is cognitive–behavioral family-based treatment (CBFT), which includes the same core components of CBT with significant family involvement. Parental involvement has shown to be a predictor relevant to improve the benefits of CBT in reducing obsessive–compulsive symptoms (11), whereas others showed that CBT for pediatric OCD is effective when delivered in different formats and that the active involvement of parents is not a crucial factor for the treatment effects (12).

Although family CBT reports high rates of remission, some children do not adequately respond to treatment to these first-line treatments, highlighting the need to identify predictors of poor response, such as comorbidity, baseline OCD severity, age at onset, parental psychopathology, behavior management skills (parental tools), family accommodation, etc. (13–16).

Predictor variables can be classified into eight broad classes (17, 18): (a) demographic variables; (b) characteristics of OCD symptoms such as severity; (c) comorbidity and associated symptom severity; (d) cognitive influences; (e) motivational factors such as treatment expectations; (f) treatment factors (e.g., compliance, therapeutic alliance); (g) biological factors; (h) other factors (e.g., family, treatment-specific characteristic).

A review of response predictors to CBT concluded there are inconsistencies in the literature (16). Therefore, this study focuses on three factors as these are considered relevant in the effectiveness of treatment of young children with OCD. These are comorbidity, initial severity, and father's and mother's accommodation.

Previous research found that pretreatment OCD severity was a negative moderator of treatment outcome, whereas comorbid anxiety was a positive moderator of treatment effect (12). Højgaard et al. (19) found that pretreatment OCD severity and levels of comorbid anxiety predict posttreatment OCD severity. However, when controlling for the effect of these predictors in a single model, only lower pretreatment OCD severity remained a significant, positive predictor of posttreatment OCD severity. Several studies showed that pretreatment OCD symptom severity significantly predicted posttreatment OCD outcome, and explained 20.6% of variance in posttreatment Yale–Brown Obsessive–Compulsive Scale (Y-BOCS) (20). Ginsburg et al. (21) showed that baseline severity of obsessive–compulsive symptoms and family dysfunction were associated with poorer response to CBT; also, comorbid tics and comorbid oppositional defiant disorder predicted poorer response to medication treatment. García et al. (22) reported that patients with higher levels of externalizing symptoms achieved worse results than those with low levels of these symptoms. Regarding depressive symptoms, some authors have found the presence of depression was not associated with a poorer response to treatment (23). Nevertheless, others showed that higher average OCD severity was associated with greater depressive symptoms across treatment but that regardless of initial depressive symptom severity, these decreased in line with reductions in OCD symptom severity (24). Other studies do not coincide with the above, concluding that pretreatment levels of symptom severity or presence of comorbidity predicted the outcome at posttreatment or at follow-up (16, 25).

Family accommodation (e.g., providing reassurance, providing items, assisting in avoidance, modifying routines) has also been studied as a predictor of response to treatment for several reasons. Mainly, high rates of accommodation among families of children with OCD suggest that up to 60–96% of relatives modify their family routines and even perform the child's rituals (22, 26–32). Second, the absence of or little introspection of minors can hinder involvement in treatment; also, the parents can participate as co-therapists and carry out exposure tasks at home, allowing us to come closer to ideal treatment as this contributes both to generalizing and maintaining achievements, and so on (5, 14, 33–36).

Some factors have been associated with parental accommodation, such as OCD symptom severity, functional impairment, child's internalizing and externalizing symptoms, parent psychopathology, and so on (5, 26–29, 36). In addition, it has been considered as a predictor of treatment outcomes (22, 37), although other studies were not found to predict outcome at posttreatment or at follow-up (25).

Given these discrepancies, the main aim of our study was to investigate the predictive factors of the initial accommodation of the father and mother of young children with OCD together with checking if accommodation and levels of externalizing disorders are variable mediators in the efficacy of treatment both at posttest and at follow-up at 6 months.

Analyzing separately the accommodation of each member of the couple is due to the fact that most studies have focused on only one involved parent (most commonly the mother) or clustered together different family members within the same study. Therefore, it would be interesting to analyze the differential response between family relatives to a child's OCD symptoms. Although focused on anxiety disorders, Thompson- Thompson-Hollands et al. (38) examined parental accommodation among mothers and fathers, concluding the mothers were slightly more accommodated than fathers. Focused on pediatric OCD, there are very few studies that have separately analyzed differences in father's and mother's accommodation. Futh et al. (39) found no differences between the two, whereas Monzani et al. (40) reported that mothers achieved significantly higher levels of accommodation than fathers. Both maternal and paternal accommodation significantly predicted worse treatment outcomes. In both studies, hierarchical analysis and regression were presented, reaching different results in children aged 7–18 years.

This research studies predictive factors in young children with OCD (5–8 years), it separately analyzes the accommodation of the father and mother, and it carries out an analysis of mediating variables of the efficacy of treatment in posttest and follow-up.

The specific objectives of the study were:

(a) To analyze the predictive factors of the initial accommodation of the father and mother, taking into account variables related to the children [severity of obsessive–compulsive responses—Children's Yale–Brown Obsessive–Compulsive Scale (CY-BOCS), internalizing and externalizing symptoms–Child Behavior Checklist, and comorbidity with other disorders] and the parents themselves in pretreatment (parental accommodation).
(b) To study whether the variables parental accommodation and externalizing symptoms would be mediating variables between initial severity and severity in obsessions and compulsions following intervention.

## Materials and Methods

### Participants

Participants were 56 children (79% males) between 5.20 and 7.67 years old (mean = 6.61 [SD = 0.76]) recruited from eight public and private clinics in Spain from 2012 to April 2021. Five clinics—psychological consultations—were private and three public (Mental Health Units for Children and Youth). All were Caucasian (white-European) and urban. The inclusion criteria were as follows: (a) primary diagnosis of OCD according to *Diagnostic and Statistical Manual of Mental Disorders, Fourth Edition, Text Revision* (*DSM-IV-TR*) and *DSM-5* criteria (41, 42); (b) a clinical severity rating of ≥16 in Children's Yale–Brown Obsessive–Compulsive Scale (43); and (c) having parents available to actively participate in the therapeutic process. Upon study entry, all parents of participating children were married and cohabitating. Exclusion criteria included (a) comorbid autism spectrum disorder, attention-deficit/hyperactivity disorder, psychotic symptoms, developmental disorder that would affect the child's ability to participate in treatment, or intellectual disability; (b) concurrent psychological therapy; and (c) medications not stable for >8 weeks. Sample characteristics are presented in Table 1.

**Table 1:** Demographic and diagnostic information.

**Characteristics**	***N* = 56**
Age (mean ± SD), years	6.61 ± 0.76
**Gender, n (%)**
Males	44 (79)
Females	12 (21)
Duration of disorder (mean ± SD), years	0.63 ± 0.20
**Comorbid diagnosis, n (%)**
Separation anxiety	8 (14.2)
Dark phobic	12 (21.4)
Animals phobic	4 (7.1)
Mood	4 (7.1)
**Comorbid diagnosis, n (%)**
One	20 (71.4)
Two	8 (28.6)
Age of mother (mean ± SD), years	36.80 ± 3.60
Age of father (mean ± SD), years	36.90 ± 3.89
**Mother education, n (%)**
Elementary	2 (3.6)
High school	13 (23.2)
College/university	41 (73.2)
**Father education, n (%)**
Elementary	1 (1.8)
High school	15 (26.8)
College/university	40 (71.4)
**Primary obsessions**
Hoarding/saving	3 (5.4)
Magical/superstitions	6 (10.6)
Aggressive	22 (39.3)
Contamination	22 (39.3)
Miscellaneous	3 (5.4)
**Primary compulsions**
Checking/reassurance	15 (26.8)
Repeating	10 (17.9)
Washing/cleaning	16 (28.6)
Involving other person	13 (23.2)
Hoarding/saving	2 (3.5)

### Procedure

**Figure 1:**
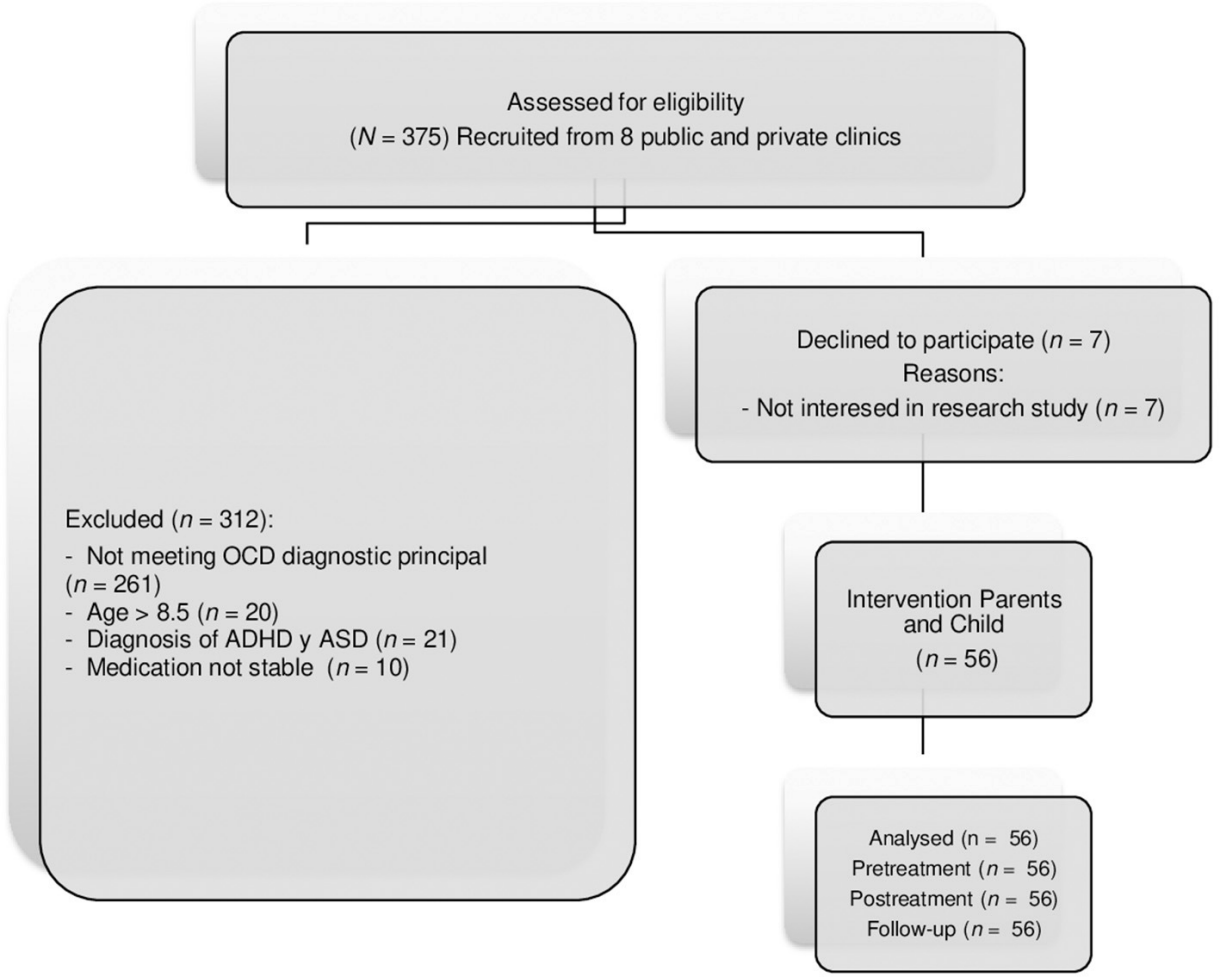
CONSORT flow diagram of study development. OCD, Obsessive-Compulsive disorder; ADHD, Attention deficit hyperactivity disorder; ASD, Autism spectrum disorder.

The study met ethical standards according to the Declaration of Helsinki and has been approved by the Ethics Committee of the University of Murcia (Spain, n°: ID: 2123/2018). All families provided written informed consent. The recruitment flow is shown in Figure 1. The procedure was as follows: (1) information from eight clinics about study performance (from 2012 to 2021); and (2) the first and the second authors carried out an unstructured clinical interview (*DSM-IV-TR* and *DSM-5*). Two specialized child psychologists administered the Initial Assessment Interviews Involved (44) and the CY-BOCS (43) to parents (3). The children with CY-BOCS ≥16 met the inclusion criteria and formed part of the program.

Assessments were done in three time points (pretreatment, posttreatment, and 6-month follow-up period) by two clinicians specialized in childhood anxiety disorders. Treatment was implemented by two clinicians specialized in OCD (over 15 years of experience). Clinicians were trained to administer CBT protocol in the same way. Degree of experience of the clinicians in Exposure Response Prevention (ERP) was the same. They applied the protocol in uniform fashion prior to initiating the study. They were doctors of clinical psychology and researchers at the OCD. Considering their years of experience, we can assume that both clinicians exhibited similar competence. In addition, both were experts in exposure with response prevention and followed the protocol indicated.

### Materials

#### Anxiety Disorders Interview Schedule for DSM-IV-Child and Parents

This interview (44) was administered to the children and parents. It has been widely used, demonstrating validity and interrater reliability in samples with very young children.

#### Children's Yale–Brown Obsessive–Compulsive Scale

This interview was jointly administered to children and parents to assess overall OCD symptom severity (43). The CY-BOCS yields an obsession severity score, compulsion severity, and total score. This scale has shown excellent psychometric properties in very young children. A total score ≥16 is considered clinically significant. In this study, Cronbach α was 0.86.

#### Child Behavior Checklist

The CBCL (45) includes 118 items to assess behavioral and emotional problems in children (externalizing and internalizing symptoms) during the prior 6 months. This measure has shown adequate internal consistency, convergent validity, and sensitivity and specificity. In this study, Cronbach α for internalizing and externalizing symptoms was high (α = 0.89; α = 0.90, respectively).

#### Family Accommodation Scale

Items are rated according to a 5-point Likert scale from 0 (never) to 4 (extreme). This instrument has shown strong internal consistency and convergent validity (46). It is considered the criterion standard in measuring family accommodation (47). In this study, Cronbach α's were 0.90 for mothers and 0.89 for fathers.

#### Penn State Worry Questionnaire

A 16-item self-report scale assesses the tendency to worry excessively about various life situations (48). The cutoff point for detection of Generalized Anxiety Disorder is 56. It has good psychometric properties. Cronbach α was high in GAD group (α = 0.96). In this study, Cronbach α's were 0.92 for mothers and 0.90 for fathers.

### Treatment

The treatment protocol was based on other programs on best practice guidelines and research evidence. This included workbooks for parents and children (49). Both parents participated in all treatment sessions. Treatment is CBFT involving 12 weekly sessions each running for 1 h and one booster session (6 months posttreatment). The workbook for children contained child-friendly examples, and the concept of OCD was presented metaphorically (i.e., an annoying ball that the child tries to throw away, but which repeatedly comes back). Ten ERP sessions were applied. A summary of the protocol can be requested from the corresponding author.

### Data Analysis

All participants were included in the analyses, with no missing cases being produced. SPSS Statistics version 23 was used for statistical analysis. A dependent-sample *t*-test was used to examine potential differences in parental accommodation.

Pearson correlations were calculated to analyze the relationship between family accommodation and other variables of the child and parents, subsequently proceeding to a hierarchical regression analysis. Mediation analyses were conducted using the PROCESS macro (50) in SPSS version 23. Mediation analyses were conducted to examine the mediating role of externalizing symptoms and the mother's accommodation in the relation between CY-BOCS pretest and CY-BOCS posttreatment and follow-up. Results reflect three analyses: (1) effect of CY-BOCS pretreatment/posttreatment on the mediator, (2) effect of mediator on the outcome of interest and effect of CY-BOCS pretreatment/posttreatment on the outcome while controlling for the mediator (direct effect), (3) the effect of CY-BOCS pretreatment/posttreatment on the outcome by way of the mediator—indirect effect. PROCESS uses a bootstrapping method that provides a confidence interval around the indirect effect, and when zero is not included in the confidence interval, this indicates a significant indirect effect. A 95% confidence interval was computed using 10,000 resamplings. All reported coefficients are unstandardized.

## Results

### Predictors of Parental Accommodation in the Pretest

To analyze the relationship before the intervention (pretreatment) between parental accommodation and variables related to the child (severity of OCD—CY-BOCS, internalizing and externalizing symptoms—CBCL, internalizing and externalizing and comorbidity) and to the parents (mother's and father's worry and mother's and father's accommodation). Bivariate Pearson correlation coefficients were calculated. Table 2 presents the results.

**Table 2:** Pearson correlation coefficients between family accommodation and other variables.

**Variable**	**Mean (SD)**	**Mother's accommodation**	**Father's accommodation**
Father's accommodation	19.00 (2.77)	0.557^**^	
Mother's accommodation	22.91 (2.77)	—	0.557^***^
Baseline CY-BOCS	22.73 (3.97)	0.740^***^	0.511^***^
CBCL-internalizing	21.09 (2.32)	0.345^*^	0.170
CBCL-externalizing	21.23 (2.79)	0.774^***^	0.520^***^
Comorbidity	2.10 (0.71)	0.226	0.017
Mother's worry	37.11 (3.55)	0.365^*^	0.281
Father's worry	27.84 (3.64)	0.249	0.401^**^

*CY-BOCS, Children's Yale–Brown Obsessive–Compulsive Scale; CBCL-internalizing/externalizing, Child Behavior Checklist*.

*** *p < 0.001*,

** *p < 0.01*,

* *p < 0.05*.

The means of father's and mother's accommodation were significantly different, highlighting greater accommodation by the mother (*t* = 10.20, *p* < 0.001). Furthermore, we observe a medium–high significant correlation between father's and mother's accommodation (*r* = 0.557). Mother's accommodation presented highest correlations with CBCL-externalizing, *r* = 0.774, and with total CY-BOCS, *r* = 0.740. The highest correlation in father's accommodation was observed with mother's accommodation, *r* = 0.557, and CBCL-externalizing, *r* = 0.520.

Subsequently, two hierarchical regression analyzes were carried out to analyze the predictive capacity of the variables of the child and the parents on accommodation. Therefore, in each regression model, the dependent variable was mother's accommodation and father's accommodation, respectively. Results are presented in Table 3.

**Table 3:** Hierarchical regression analysis for predictors of mother's and father's accommodation.

**Dependent variable**	**Predictors**	$R_{\text{adj}}^{2}$	***b_***j***_***	***t***	***p***
**Mother's accommodation**	**Step 1:**				
	CBCL-externalizing	0.590	0.774	7.927	<0.001
	**Step 2:**				
	CBCL-externalizing	0.687	0.510	4.604	<0.001
	Pretreatment total CYBOCS		0.415	3.748	0.001
**Father's accommodation**	**Step 1:**				
	Mother's accommodation	0.294	0.557	4.347	<0.001
	**Step 2:**				
	Mother's accommodation	0.354	0.487	3.849	<0.001
	Father's worry		0.280	2.209	0.033

*CBCL-internalizing/externalizing, Child Behavior Checklist; CY-BOCS, CY-BOCS, Children's Yale–Brown Obsessive–Compulsive Scale. R${}_{\text{adj}}^{2}$, adjusted coefficient of determination (proportion of variance explained by the model); b_j_, partial regression coefficient associated with each predictor of the model; tm t-test to contrast the significance of statistical significance of each predictor in the model; p, probability level associated with the t-test*.

CBCL-externalizing and total CY-BOCS in pretreatment explained 68.7% of variance. The positive sign of the regression coefficients revealed the existence of a direct relationship with mother's accommodation.

Mother's accommodation and father's worry in the pretreatment explained 35.4% of explained variance. The positive sign of their regression coefficients indicated a direct relationship between them.

### Mediator Models on the Efficacy of Treatment

#### CBCL, Mediator Variable in Obsessive–Compulsive Severity in Posttreatment and Follow-Up

This model evaluated whether externalizing symptoms pretest would mediate the relationship between initial obsessive–compulsive severity (CY-BOCS pretest) and CY-BOCS posttreatment. Table 4 shows that CBCL externalizing pretest significantly mediated the relationship between CY-BOCS posttreatment as the bootstrap confidence interval (CI) was greater than zero. The total effect of CY-BOCS pretest on CY-BOCS posttreatment was significant (CI = 0.28–0.46, *t* = 8.31, *p* < 0.001). Meanwhile, the regression coefficient estimates, based on the use of 95% bias-corrected CI as evidence of the mediation of total indirect was 0.094 (CI = 0.014–0.195). The CBCL-externalizing posttreatment significantly mediated the relationship between CY-BOCS follow-up as the bootstrap CI was greater than zero. The total effect of CY-BOCS posttreatment on CY-BOCS follow-up was significant (CI = 0.428–0.670, *t* = 9.150, *p* < 0.001).

**Table 4:** Association between CY-BOCS, as mediated by CBCL externalizing.

**Outcome variable**	**Predictor**	**Coefficient (SE)**	**CI**	***t***	***p***
CBCL-ext. pretreatment	CY-BOCS pretreatment	0.45 (0.08)	0.28–0.62	5.36	<0.000
CY-BOCS posttreatment	CBCL-ext. pretreatment	0.21 (0.08)	0.06–0.34	2.76	0.008
	CY-BOCS pretreatment	0.27 (0.05)	0.17–0.38	5.13	<0.000
	**Indirect effect**	**Effect (boot SE)**	**CI**		
CY-BOCS posttreatment	CBCL-ext. pretreatment				
	0.094	0.05	0.01–0.20		
CBCL-ext. posttreatment	CY-BOCS follow-up	1.41 (0.23)	0.94–1.88	6.09	<0.001
CY-BOCS follow-up	CBCL-ext. posttreatment	0.11 (0.04)	0.31–1.80	2.85	0.006
	CY-BOCS posttreatment	0.40 (0.08)	0.25–0.55	5.26	<0.001
	**Indirect effect**	**Effect (boot SE)**	**CI**		
CY-BOCS follow-up	CBCL-ext posttreatment				
	0.15	0.06	0.05–0.26		

*CBCL-Ext, Child Behavior Checklist externalizing; CY-BOCS, Children's Yale–Brown Obsessive–Compulsive Scale. t = t-test to contrast the significance of statistical significance of each predictor in the model; p = probability level associated with the t-test; CI = 95% bootstrap lower and upper limit confidence interval*.

#### Mother's Accommodation, Mediator Variable in Obsessive–Compulsive Severity in Posttreatment and Follow-Up

This model evaluated whether accommodation pretest would mediate the relationship between initial severity of obsessive–compulsive responses (CY-BOCS pretreatment). Table 5 reports that mother's accommodation pretreatment and posttreatment did not significantly mediate the relationship between CY-BOCS posttreatment and follow-up because the bootstrap CI was not greater than zero. The total effect of CY-BOCS pretest on CY-BOCS posttreatment was significant (CI = 0.278–0.457, *t* = 8.308, *p* < 0.001). The total effect of CY-BOCS posttreatment on CY-BOCS follow-up was significant (CI = 0.428–0.670, *t* = 9.15, *p* < 0.001).

**Table 5:** Association between CY-BOCS, as mediated by mother's accommodation.

**Outcome variable**	**Predictor**	**Coefficient (SE)**	**CI**	***t***	***p***
Mother's accommodation pretreatment	CY-BOCS pretreatment	0.49 (0.07)	0.35–0.63	7.13	<0.000
CY-BOCS posttreatment	Mother's accommodation pretreatment	0.20 (0.10)	0.02–0.392	2.04	042
	CY-BOCS pretreatment	0.2702 (0.0603)	0.14–0.40	4.29	001
	**Indirect effect**	**Effect (boot SE)**	**CI**		
CY-BOCS posttreatment	Mother's accommodation pretreatment				
	0.096	0.05	−0.04 to 0.17		
Mother's accommodation posttreatment	CY-BOCS posttreatment	1.21 (0.21)	0.79–1.64	5.75	<0.001
CY-BOCS follow-up	Mother's accommodation posttreatment	0.05 (0.44)	−0.03 to 0.14	1.23	0.227
	CY-BOCS posttreatment	0.48 (0.08)	0.32–0.65	6.07	<0.000
	**Indirect effect**	**Effect (boot SE)**	**CI**		
CY-BOCS follow-up	Mother's accommodation posttreatment				
	0.065	0.04	−0.02 to 0.14		

## Discussion

Pediatric OCD is a debilitating psychological condition associated with interference in child's recreational, academic, social, and family activities (1). Various studies have been conducted to enhance the efficacy of treatments and predictive variables (18, 22, 47). However, we are not aware of any study focused on the analysis of predictors and mediators of treatment in young children (5–8 years) with OCD.

One of the main aims of our study was to investigate the predictive factors of the initial mother's and father's accommodation of young children with OCD. Many researchers have studied family accommodation, reporting a high implication in the efficacy of treatment (22, 37). The age of our sample led us to suppose that parents would be highly accommodated with their children's rituals. These are young children whose main manifestation will be compulsions and rituals that will involve their parents. The results of the current study have indicated a high correlation between obsessive–compulsive responses and accommodation of the mother and father, consistent with previous studies (24, 39, 40). We have been able to observe that the mother's accommodation was greater than the father's, coinciding with the study of Monzani et al. (40), which might simply relate to the amount of time a caregiver spends with a child with OCD. The mothers spent a greater number of hours [mean = 10 (SD = 2.7) h] than the fathers [mean = 5 (SD = 4.58) h]. Furthermore, a high correlation between accommodation mother and father scores was also observed, suggesting that if one parent accommodates a child's symptoms, the other seems more likely to do so as well.

The results of the hierarchical regression analysis indicated that the mother's accommodation could be predicted according to the child variables CBCL-externalizing and initial OCD severity (CY-BOCS pretest), whereas the father's accommodation could be better explained by variables mother's accommodation and father's worry. The results of the mother's accommodation coincide with those indicated by Caporino et al. (26), although the percentage of variance explained in our study was higher (68.7%).

The externalizing symptoms of the child would be the main predictor explaining 59% of variance. Children who present externalizing symptoms can cause family conflict, difficulty in the relationship with parents, punishments, and so on, being more resistant in carrying out treatment. Parents' abilities to manage their children's disruptive behaviors would be an important variable when it comes to better understanding the efficacy of treatment. This result is in line with other research as mothers accommodate themselves in order to avoid the child's problem behaviors, thus increasing the child's compulsive behaviors (40). In addition, the severity symptoms obsessive–compulsive predicted maternal accommodation, being somewhat expected and coinciding with that reported in previous studies (5, 7, 51, 52). Therefore, mothers of children with greater behavioral problems and greater severity of the disorder will present greater accommodation. Therefore, externalizing problems and comorbidity with externalizing disorders must be taken into account when carrying out treatment in children with OCD. In addition, the severity symptoms obsessive–compulsive predicted maternal accommodation, being somewhat expected and coinciding with that reported in previous studies (5, 7, 51, 52). Therefore, mothers of children with greater behavior problems and greater severity of the disorder will present greater accommodation.

Father's accommodation was not explained by the severity of the obsessive–compulsive behaviors but by the mother's accommodation and the father's worry. The percentage explained by both predictors was lower (35.4%). One possible explanation is that the mother's coping responses influence the way in which the father intervenes with the child when obsessive–compulsive responses occur. This could be due to the fact that the mother spends most of the time with the child, producing the main changes. On the other hand, parents with greater worry and anxiety increased their accommodation responses, as these temporarily reduced the fear and worry. This result is in line with that reported by Monzani et al. (40) in which father's distress was associated with mother's accommodation. These results would inform a circle that would maintain and enhance maintenance of the child's problem responses (26).

Another of our aims was to study whether the variables parental accommodation and externalizing disorders would be mediating variables between initial severity and externalizing symptoms, which were mediators between the initial and posttest severity levels, although the value of the coefficient was small. Similarly, these problems mediated results at follow-up. This result emphasized the importance of comorbidities, specifically externalizing symptoms, in treatment results, consistent with previous research (21, 22). In addition, our results highlight the potential value of screening and providing additional support for parents of children with obsessive disorders and externalizing symptoms in order to adapt treatment to both types of symptoms.

Mother's accommodation was not a mediating factor between initial severity and posttreatment and follow-up severity. Its relationship with severity pretreatment and posttreatment was observed, but could not be considered a mediator in improvement after intervention. This indicates that the main moderator to take into account to reduce obsessive–compulsive symptoms was the external symptoms of the child. Therefore, it is necessary to train in this type of problem to improve obsessive–compulsive symptoms (5, 51).

Clinical implications of this study are the need to take into account not only mothers in treatment but also fathers as they can be indirectly influenced. We can conclude that the comorbidities of externalizing symptoms, the father's worry, and the mother's accommodation are variables that we should control for when performing a treatment in pediatric OCD. Emerging evidence supports the benefits of family-based CBT, incorporating treatment modules that address externalizing symptoms and family accommodation. Sukhodolsky et al. (53) analyzed the importance of including the intervention together with the ERP training for parents in the management of disruptive behaviors, achieving greater improvement when both strategies were combined in children with secondary diagnosis of problems of conduct. Rosa-Alcázar et al. (49) indicated that the involvement of both parents in the child's treatment showed statistically significant differences in internalizing and externalizing symptoms and mother's and father's accommodation.

There are several study limitations. First, the small sample size limits the generalizability of our results. Second, follow-up outcomes are insufficient; that is, they have a provisional nature, and follow-up is required at 12 and 24 months to more rigorously assess the scope of achievement stability. Third, the study relied on parents as central informants, and no measures were in place to ensure that mothers' and fathers' responses were collected independently from the other parent. Finally, causality between accommodation and treatment response cannot be established from this study. More research is needed to confirm the direction of causation in order to inform intervention strategies.

Future research should examine not only the efficacy of these additional treatment components but also in what sequence these additional components should be presented for young people with OCD and comorbidity with externalizing symptoms, as indicated by other authors (22). The inclusion of more predictors, increasing the sample size, could help to better analyze the effects of mediation on treatment results in young children with OCD.

Notwithstanding these limitations, the analysis of the predictors of mother's and father's accommodation in the pretreatment together with the study of a mediation model to clarify the relationships between severity pretreatment and posttreatment/follow-up and externalizing symptoms and mother's accommodation is an important contribution to the pediatric OCD literature.

## Data Availability Statement

The raw data supporting the conclusions of this article will be made available by the authors, without undue reservation.

## Ethics Statement

The studies involving human participants were reviewed and approved by Ethics Committee of Murcia University. Written informed consent to participate in this study was provided by the participants' legal guardian/next of kin.

## Author Contributions

AR-A and ÁR-A: conceptualization. AR-A, ÁR-A, PO-O, JP-N, and ER-A: treatment, writing—original draft preparation, review, and editing. AR-A: methodology, formal analysis, data curation, and supervision. All authors have read and agreed to the published version of the manuscript.

## Funding

This research is part of Project 20902/PI/18 financed by the Autonomous Community of Murcia (Spain) through the Grants for projects for the development of scientific and technical research by competitive groups, included in the Regional Program for the Promotion of Scientific and Technical Research (Action Plan 2018) of the Seneca Foundation-Science and Technology Agency of Murcia Region (Spain).

## Conflict of Interest

The authors declare that the research was conducted in the absence of any commercial or financial relationships that could be construed as a potential conflict of interest.

## Publisher's Note

All claims expressed in this article are solely those of the authors and do not necessarily represent those of their affiliated organizations, or those of the publisher, the editors and the reviewers. Any product that may be evaluated in this article, or claim that may be made by its manufacturer, is not guaranteed or endorsed by the publisher.

## References

[1] Valderhaug R Ivarsson T. Functional impairment in clinical samples of Norwegian and Swedish children and adolescents with obsessive-compulsive disorder. Eur Child Adolesc Psychiatry. (2005) 14:164–73. 10.1007/s00787-005-0456-9 15959662

[2] Geller DA March J. Practice parameter for the assessment and treatment of children and adolescents with obsessive-compulsive disorder. Focus. (2012) 10:360–73. 10.1176/appi.focus.10.3.360 22176943

[3] Heyman I Fombonne E Simmons H Ford T Meltzer H Goodman R. Prevalence of obsessive-compulsive disorder in the British nationwide survey of child mental health. Int Rev Psychiatry. (2003) 15:178–84. 10.1080/0954026021000046146 12745330

[4] James SC Farrell LJ Zimmer-Gembeck MJ. Description and prevalence of OCD in children and adolescents. In: Abramowitz JS, McKay D, Storch EA, editors. Handjournal of Obsessive-Compulsive Disorder Across the Life Span. New York, NY: Wiley (2017). p. 5–23. 10.1002/9781118890233.ch1

[5] Wu MS Storch EA. Personalizing cognitive-behavioral treatment for pediatric obsessive-compulsive disorder. Expert Rev Precis Med Drug Dev. (2016) 1:397–405. 10.1080/23808993.2016.1209972

[6] Storch EA Merlo LJ Larson MJ Geffken GR Lehmkuhl HD Jacob ML. Impact of comorbidity on cognitive-behavioral therapy response in pediatric obsessive-compulsive disorder. J Am Acad Child Adolesc Psychiatry. (2008) 47:583–92. 10.1097/CHI.0b013e31816774b1 18356759

[7] Lavell CH Farrell LJ Waters AM Cadman J. Predictors of treatment response to group cognitive behavioural therapy for pediatric obsessive-compulsive disorder. Psychiatry Res. (2016) 245:186–93. 10.1016/j.psychres.2016.08.033 27544784

[8] Murray K Jassi A Mataix-Cols D Barrow F Krebs G. Outcomes of cognitive behaviour therapy for obsessive–compulsive disorder in young people with and without autism spectrum disorders: a case controlled study. Psychiatry Res. (2015) 228:8–13. 10.1016/j.psychres.2015.03.012 25935374

[9] Storch EA Lewin AB Geffken GR Morgan JR Murphy TK. The role of comorbid disruptive behavior in the clinical expression of pediatric obsessive-compulsive disorder. Behav Res Ther. (2010) 48:1204–10. 10.1016/j.brat.2010.09.004 20933220

[10] Selles RR Belschner L Negreiros J Lin S Schuberth D McKenney K. Group family-based cognitive behavioral therapy for pediatric obsessive compulsive disorder: Global outcomes and predictors of improvement. Psychiatry Res. (2018) 260:116–22. 10.1016/j.psychres.2017.11.041 29179016

[11] Rosa-Alcázar AI Iniesta-Sepúlveda M Storch EA Rosa-Alcázar Á Parada-Navas JL Rodríguez JO. A preliminary study of cognitive-behavioral family-based treatment versus parent training for young children with obsessive-compulsive disorder. J Affect Disord. (2017) 208:265–71. 10.1016/j.jad.2016.09.060 27792972

[12] Öst LG Riise EN Wergeland GJ Hansen B Kvale G. Cognitive behavioral and pharmacological treatments of OCD in children: a systematic review and meta-analysis. J Anxiety Disord. (2016) 43:58–69. 10.1016/j.janxdis.2016.08.003 27632568

[13] Du Mortier JA Remmerswaal KC Batelaan NM Visser HA Twisk JW van Oppen P. Predictors of Intensive treatment in patients with obsessive-compulsive disorder. Front Psychiatry. (2021) 12:434. 10.3389/fpsyt.2021.659401 33912087 PMC8072047

[14] Lebowitz ER. Treatment of extreme family accommodation in a youth with obsessive-compulsive disorder. In: Storch AE, Lewin AB, editors. Clinical Handjournal of Obsessive-Compulsive and Related Disorders. Switzerland: Sprinter International Publishing Switzerland (2016). p. 321–35. 10.1007/978-3-319-17139-5_22

[15] McGuire JF Piacentini J Lewin AB Brennan EA Murphy TK Storch EA. A meta-analysis of cognitive behavior therapy and medication for child obsessive-compulsive disorder: Moderators of treatment efficacy, response, and remission. Depress Anxiety. (2015) 32:580–93. 10.1002/da.22389 26130211 PMC4515191

[16] Olatunji BO Davis ML Powers MB Smits JA. Cognitive-behavioral therapy for obsessive-compulsive disorder: A meta-analysis of treatment outcome and moderators. J Psychiatr Res. (2013) 47:33–41. 10.1016/j.jpsychires.2012.08.020 22999486

[17] Keeley ML Storch EA Merlo LJ Geffken GR. (2008). Clinical predictors of response to cognitive-behavioral therapy for obsessive–compulsive disorder. Clin Psychol Rev. (2008) 28:118–30. 10.1016/j.cpr.2007.04.003 17531365

[18] Kyrios M. Exposure and response prevention in the treatment of obsessive-compulsive disorder. In: Menzies R, editor. Obsessive-Compulsive Disorder: Theory, Research and Treatment Chichester DSP. England: John Wiley & Sons (2003). p. 259–74.

[19] Højgaard DR Hybel KA Mortensen EL Ivarsson T Nissen JB Weidle B. Obsessive-compulsive symptom dimensions: association with comorbidity profiles and cognitive-behavioral therapy outcome in pediatric obsessive-compulsive disorder. Psychiatry Res. (2018) 270:317–23. 10.1016/j.psychres.2018.09.054 30290317

[20] Kyrios M Hordern C Fassnacht DB. Predictors of response to cognitive behaviour therapy for obsessive-compulsive disorder. Int J Clin Health Psychol. (2015) 15:181–90. 10.1016/j.ijchp.2015.07.003 30487835 PMC6225019

[21] Ginsburg GS Kingery JN Drake KL Grados MA. Predictors of treatment response in pediatric obsessive-compulsive disorder. J Am Acad Child Adolesc Psychiatry. (2008) 47:868–78. 10.1097/CHI.0b013e3181799ebd 18596553

[22] Garcia AM Sapyta JJ Moore PS Freeman JB Franklin ME March JS. Predictors and moderators of treatment outcome in the Pediatric Obsessive Compulsive Treatment Study (POTS I). J Am Acad Child Adolesc Psychiatry. (2010) 49:1024–33. 10.1016/j.jaac.2010.06.013 20855047 PMC2943932

[23] Farrell LJ Waters AM Zimmer-Gembeck MJ. Cognitive biases and obsessive-compulsive symptoms in children: examining the role of maternal cognitive bias and child age. Behav Ther. (2012) 43:593–605. 10.1016/j.beth.2011.10.003 22697447

[24] Meyer J M McNamara JP Reid AM Storch EA Geffken GR Mason DM. Prospective relationship between obsessive–compulsive and depressive symptoms during multimodal treatment in pediatric obsessive–compulsive disorder. Child Psychiatry Hum Dev. (2014) 45:163–72. 10.1007/s10578-013-0388-4 23756717

[25] Riise EN Kvale G Öst LG Skjold SH Hansen B. Does family accommodation predict outcome of concentrated exposure and response prevention for adolescents? Child Psychiatry Hum Dev. (2019) 50:975–86. 10.1007/s10578-019-00898-1 31134420

[26] Caporino NE Morgan J Beckstead J Phares V Murphy TK Storch EA. A structural equation analysis of family accommodation in pediatric obsessive-compulsive disorder. J. Abnorm Child Psychol. (2012) 40:133–43. 10.1007/s10802-011-9549-8 21842196

[27] Flessner CA Sapyta J Garcia A Freeman JB Franklin ME Foa E. Examining the psychometric properties of the family accommodation scale-parent-report (FAS-PR). J Psychopathol Behav Assess. (2011) 33:38–46. 10.1007/s10862-010-9196-3 21743772 PMC3131184

[28] Lebowitz ER Scharfstein LA Jones J. Comparing family accommodation in pediatric obsessive-compulsive disorder, anxiety disorders, and nonanxious children. Depress Anxiety. (2014) 31:1018–25. 10.1002/da.22251 24677578

[29] Merlo LJ Lehmkuhl HD Geffken GR Storch EA. Decreased family accommodation associated with improved therapy outcome in pediatric obsessive–compulsive disorder. J Consult Clin Psychol. (2009) 77:355. 10.1037/a0012652 19309195 PMC2886196

[30] Shafran R Ralph J Tallis F. Obsessive-compulsive symptoms and the family. Bull Menninger Clin. (1995) 59:472. 8535386

[31] Stewart SE Beresin C Haddad S Egan Stack D Fama J Jenike M. Predictors of family accommodation in obsessive-compulsive disorder. Ann Clin Psychiatry. (2008) 20:65–70. 10.1080/10401230802017043 18568577

[32] Storch EA Geffken GR Merlo LJ Jacob ML Murphy TK Goodman WK. Family accommodation in pediatric obsessive–compulsive Disorder. J Clin Child Adolesc Psychol. (2007) 36:207–16. 10.1080/15374410701277929 17484693

[33] Pinto A Van Noppen B Calvocoressi L. Development and preliminary psychometric evaluation of a self-rated version of the family accommodation scale for obsessive-compulsive disorder. J Obsessive Compuls Relat Disord. (2013) 457:65. 10.1016/j.jocrd.2012.06.001 24855596 PMC4024376

[34] Pontillo M Demaria F Tata MC Averna R Gargiullo P Pucciarini ML. Clinical significance of family accommodation and parental psychological distress in a sample of children and adolescents with obsessive-compulsive disorder aged 8-17 years old. Ital J Pediatr. (2020) 46:1–10. 10.1186/s13052-020-00932-2 33168039 PMC7654062

[35] Stewart SE Hu YP Leung A Chan E Hezel DM Lin SY. A multisite study of family functioning impairment in pediatric obsessive-compulsive disorder. J Am Acad Child Adolesc Psychiatry. (2017) 56:241–9. 10.1016/j.jaac.2016.12.012 28219490 PMC5332162

[36] Wu MS Geller DA Schneider SC Small BJ Murphy TK Wilhelm S. Comorbid psychopathology and the clinical profile of family accommodation in pediatric OCD. Child Psychiatry Hum Dev. (2019) 50:717–26. 10.1007/s10578-019-00876-7 30790098 PMC6703960

[37] Torp NC Dahl K Skarphedinsson G Compton S Thomsen PH Weidle B. Predictors associated with improved cognitive-behavioral therapy outcome in pediatric obsessive-compulsive disorder. J Am Acad Child Adolesc Psychiatry. (2015) 54:200–7. 10.1016/j.jaac.2014.12.007 25721185

[38] Thompson-Hollands J Edson A Tompson MC Comer JS. Family involvement in the psychological treatment of obsessive–compulsive disorder: a meta-analysis. J Fam Psychol. (2014) 28:287. 10.1037/a0036709 24798816 PMC4086156

[39] Futh A Simonds LM Micali N. Obsessive-compulsive disorder in children and adolescents: parental understanding, accommodation, coping and distress. J Anxiety Disord. (2012) 26:624–32. 10.1016/j.janxdis.2012.02.012 22440392

[40] Monzani B Vidal-Ribas P Turner C Krebs G Stokes C Heyman I. et al. The role of paternal accommodation of paediatric OCD symptoms: patterns and implications for treatment outcomes. J Abnorm Child Psychol. (2020) 48:1313–23. 10.1007/s10802-020-00678-9 32683586 PMC7445192

[41] American Psychiatric Association. Diagnostic and Statistical Manual of Mental Disorders (DSM-IV-TR). Washington, DC: American Psychiatric Association (2000).

[42] American Psychiatric Association. Diagnostic and Statistical Manual of Mental Disorders (DSM-5). Washington, DC: American Psychiatric Association (2013). 10.1176/appi.journals.9780890425596

[43] Scahill L Riddle MA McSwiggin-Hardin M Ort SI King RA Goodman WK. Children's Yale-Brown obsessive–compulsive scale: reliability and validity. J Am Acad Child Adolesc Psychiatry. (1997) 36:844–52. 10.1097/00004583-199706000-00023 9183141

[44] Silverman WK Albano AM Sandín B. ADIS-IV/C: Entrevista para el diagnóstico de os trastornos de ansiedad en niños según el DSM-IV: entrevista para el niño. Madrid: UNED (2003).

[45] Achenbach TM. Manual for the Child Behavior Checklist/4-18 and 1991 Profile. Burlington, VT: Department of Psychiatry, University of Vermont (1991).

[46] Calvocoressi L Lewis B Harris M Trufan SJ. Family accommodation in obsessive–compulsive disorder. Am J Psychiatry. (1995) 152:441–3. 10.1176/ajp.152.3.441 7864273

[47] Francazio SK Flessner CA Boisseau CL Sibrava NJ Mancebo MC Eisen JL. Parental accommodation predicts symptom severity at long-term follow-up in children with obsessive-compulsive disorder: A preliminary Investigation. J Child Fam Stud. (2016) 25:2562–70. 10.1007/s10826-016-0408-7 28989268 PMC5627772

[48] Meyer TJ Miller ML Metzger RL Borkovec TD. Development and validation of the Penn State Worry Questionnaire. Behav Res Ther. (1990) 28:487–95. 10.1016/0005-7967(90)90135-6 2076086

[49] Rosa-Alcázar Á Rosa-Alcázar AI Olivares-Olivares PJ Parada-Navas JL Rosa-Alcázar E Sánchez-Meca J. (2019). Family involvement and treatment for young children with Obsessive-Compulsive Disorder: Randomized control study. Int J Clin Health Psychol. (2019) 19:218–27. 10.1016/j.ijchp.2019.06.001 31516500 PMC6732770

[50] Hayes AF. Introduction to Mediation, Moderation, and Conditional Process Analysis: A Regression Based Approach. New York, NY: Guilford Press (2013).

[51] Piacentini J Bergman RL Chang S Langley A Peris T Wood JJ. Controlled comparison of family cognitive behavioral therapy and psychoeducation/relaxation training for child obsessive-compulsive disorder. J Am Acad Child Adolesc Psychiatry. (2011) 50:1149–61. 10.1016/j.jaac.2011.08.003 22024003 PMC3205429

[52] Iniesta-Sepúlveda M Rosa-Alcázar AI Sánchez-Meca J Parada-Navas JL Rosa-Alcázar Á. Cognitive-behavioral high parental involvement treatments for pediatric obsessive-compulsive disorder: A meta-analysis. J Anxiety Disord. (2017) 49:53–64. 10.1016/j.janxdis.2017.03.010 28431305

[53] Sukhodolsky DG Gorman BS Scahill L Findley D McGuire J. Exposure and response prevention with or without parent management training for children with obsessive-compulsive disorder complicated by disruptive behavior: a multiple-baseline across-responses design study. J Anxiety Disord. (2013) 27:298–305. 10.1016/j.janxdis.2013.01.005 23602943

